# Diversity of upper respiratory tract infections and prevalence of *Streptococcus pneumoniae* colonization among patients with fever and flu-like symptoms

**DOI:** 10.1186/s12879-018-3662-z

**Published:** 2019-01-07

**Authors:** Jialiang Tang, Jinkun Chen, Tingting He, Zhuojing Jiang, Jiale Zhou, Bin Hu, Shangxin Yang

**Affiliations:** 1 Shaoxing Center for Disease Control and Prevention, Shaoxing, Zhejiang, China; 2 IngeniGen XunMinKang Biotechnology Inc. Shaoxing, Zhejiang, China; 3 Zhejiang-Californina International Nanosystems Institute, Zhejiang University, Zhejiang, Hangzhou China; 4 Clinical Microbiology Laboratory, Department of Pathology and Laboratory Medicine, University of California Los Angeles, 11633 San Vicente Blvd, Los Angeles, CA 90049 USA

**Keywords:** Flu-like symptoms, Upper respiratory tract infections, Respiratory pathogens, *Streptococcus pneumoniae* colonization, Molecular testing

## Abstract

**Background:**

Many upper respiratory pathogens cause similar symptoms. In China, routine molecular tests for upper respiratory pathogens are not widely performed and antibiotics abuse in treating upper respiratory tract infections (URTIs) is a major public health concern.

**Methods:**

We performed qualitative real-time PCR tests to detect common upper respiratory tract pathogens including 9 viruses and 3 bacteria in 1221 nasopharyngeal swabs from patients with fever and influenza-like symptoms in a Chinese city. A quantitative real-time PCR was also performed to measure the bacterial density of the colonizing *Streptococcus pneumoniae* in these samples.

**Results:**

We found very diverse pathogens including 81.7% viruses, 11.6% bacteria and 6.7% mixed viruses and bacteria. *S. pneumoniae* colonization was found in 8.0% of the cases but most of them had low bacterial density (Mean = 3.9 log cfu/ml). We also discovered an increase of *S. pneumoniae* colonization frequency (but not the density) in patients with detectable upper respiratory tract pathogens, in a pathogen variety-dependent manner.

**Conclusions:**

Our study provided strong evidence against empiric antibiotic use for treating URTIs, and highlighted a strong need for improving the diagnostic capacity for URTIs by using more molecular testing in China.

## Key points

Very diverse upper respiratory pathogens with viruses as the majority were detected by real-time PCR in patients with fever and flu-like symptoms in Eastern China. *Streptococcus pneumoniae* colonization frequency, but not the density, was found to increase in patients with detectable upper respiratory pathogens, in a pathogen variety-dependent manner.

## Background

It has been well recognized that many pathogens, mainly viruses and some bacteria, cause URTIs but present with almost indistinguishable clinical symptoms [1–3]. Differentiation of viral URTIs versus bacterial URTIs has the direct implication of antibiotics use. However, a lack of timely diagnosis by using rapid and accurate tests had contributed to overuse and abuse of antibiotics around the world [4]. In China, antibiotics abuse is very common due to a culture of self-medication (antibiotics are largely available over the counter) and over-prescription by clinicians [5, 6]. Antibiotics are mainly prescribed empirically, not based on microbiological investigation [6, 7]. This is particularly problematic for patients with URTIs, for which most clinics and many hospitals in China only offer limited routine tests for the influenza viruses (Flu). Common upper respiratory tract pathogens other than Flu, such as human rhinovirus (hRV), respiratory syncytial virus (RSV), parainfluenza viruses (PIVs), adenovirus (ADV), human metapneumovirus (hMPV) and bacterial pathogens that are difficult to culture including *Mycoplasma penumoniae*, *Chlamydophila pneumoniae* and *Bordetella pertussis* are generally not tested in China, except for a few high-ranking academic medical centers (facts based on personal observations and communication with colleagues in China). Therefore, improving the accurate diagnosis of pathogens causing the URTIs (abbreviated as URTI pathogens in this article) is of great significance for rational selection of antibiotics and reduction of antibiotics abuse in China.

As one of the most densely populated provinces in China, Zhejiang has been the center of attention for emerging infectious diseases since the H7N9 bird flu outbreak first started there in early 2013 [8]. Since then, Chinese state and local government had implemented surveillance programs to actively monitor the circulating flu viruses. In one of such programs, hospitals and clinics routinely submit nasopharyngeal swabs of patients with fever and flu-like symptoms to the local public health laboratories for flu viruses testing by PCR and then genotyping by Sanger sequencing if positive. However, even during the peak of flu season, there are still many patients with fever and flu-like symptoms but tested negative for flu viruses. The question remains as what other respiratory pathogens are circulating and causing the similar flu-like symptoms, and what pathogens co-infect with flu virus in the Chinese community. Another important question is about the frequencies of bacterial pathogens such as *M. pneumoniae, C. pneumoniae* and *B. pertussis* that do require antibiotics treatment and how often they co-infect with viral pathogens. In addition, upper respiratory tract colonization of *Streptococcus pneumoniae,* the most common cause of bacterial pneumonia, was considered prerequisite for its infections in the lower respiratory tract [9]. Both the density and frequency of *S. pneumoniae* in the upper respiratory tract had been shown to increase during viral infections [10], but very limited data exist regarding the prevalence and density of *S. pneumoniae* colonization in patients with flu-like symptoms. A better understanding of the epidemiology of *S. pneumoniae* colonization and its relationship with the pathogens causing URTIs may help solve the controversy over using antibiotic prophylaxis to prevent possible secondary lower respiratory tract infections (LRTIs) caused by *S. pneumoniae*, which is considered to be another inappropriate antibiotic prescription practice that could contribute to antibiotic resistance [11, 12].

To help answer these questions, we carried out a study based on nasopharyngeal swabs collected from patients of all ages (majority adults) with URTIs in Shaoxing, the third largest city in Zhejiang province in Eastern China, in a full year (2016). We tested these upper respiratory samples for common viral URTI pathogens including FluA, FluB, RSV, hRV, PIVs (1–3), ADV, and hMPV, and common bacterial URTI pathogens including *M. pneumoniae, C. pneumoniae and B. pertussis*, as well as colonizing *S. pneumoniae* to study its relationship with these URTI pathogens.

## Methods

### Patients and clinical specimens

From January 1, 2016 to December 31, 2016, nasopharyngeal swabs were collected from 1221 outpatients (age ranged from 5 months to 99 years) with fever (≥ 38 °C) and influenza-like symptoms such as cough, runny or stuffy nose, sore throat, muscle aches, chills and fatigue. All samples were de-identified to protect patients’ private information except for the age. No clinical information such as X-ray results, severity of the illness, diagnosis, or use of antibiotics were available. The nasopharyngeal swabs were collected using the flock swabs (Copan, Italy) and stored in the Universal Transportation Medium (Copan, Italy) provided in the same collection kit. The samples were refrigerated for up to 1 week until being tested.

### Detection of respiratory pathogens by real-time PCR

Total nucleic acids were extracted using IngeniGen Total Nucleic Acids Extraction Kit, following the manufacturer’s instruction (IngeniGen XunMinKang Biotechnology Inc., Shaoxing, China), and the real-time PCR assays were performed using the IngeniGen Respiratory Pathogen Multiplex PCR Kits and ABI 7500 system (ThermoFisher, Boston, MA, USA). MS2 phage and plasmids containing the human albumin gene were added to the samples as internal controls for RNA virus detection, and DNA virus and bacteria detection, respectively.

### Quantitative real-time PCR for S. pneumoniae

The *S. pneumoniae* quantitative real-time PCR kit was purchased from IngeniGen XunMinKang Biotechnology Inc., Shaoxing, China. Included in the kit, a set of calibrators, which were DNA extracted from 10-fold serial dilutions of a laboratory strain *S. pneumoniae* (ATCC 49691) with the highest concentration of 5 × 10^6^ colony forming units per milliliter (cfu/ml) and the lowest concentration of 5 × 10^2^ cfu/ml, were tested to create a standard curve and a formula for the bacterial load quantification. The lower limit of detection (LOD) and the lower limit of quantification (LOQ) of the test were both 500 cfu/ml according to the manufacturer’s package insert. The quantification formula was determined to be Log10 cfu/ml = (49.145 - Ct value) / 3.404.

### Statistical analysis

All data were statistically processed using SPSS 22.0 and GraphPad Prism 5. The frequencies of URTI pathogens and *S. pneumoniae* colonization were analyzed using Pearson’s chi-square test (χ2) to identify any difference between a specific age group vs. all other age groups. The colonizing *S. pneumoniae* density were analyzed by one-way analysis of variance (ANOVA) to identify any difference between a specific age group vs. all other age groups, and by t-test to compare the samples with or without URTI pathogens detected. All the *S. pneumoniae* density results were summarized using means and standard deviation (SD). *P* < 0.05 was considered as statistically significant.

## Results

There were 1221 participants recruited into the study, with 71–200 recruited each month. All participants agreed to contribute the residual nasopharyngeal swab samples for this study after the routine Flu A/B PCR test was done. The additional test results were only for epidemiological research purpose and not provided to the patients. Among all age groups, the overall positive rate for the URTI pathogens was 30.5% (372/1221). The most frequently detected URTI viruses were FluA (15.7%), FluB (3.2%), RSV (3.6%) and hRV (3.4%) and the most frequently detected URTI bacteria was *M. pneumoniae* (5.3%). The other URTI pathogens including ADV, PIVs, hMPV, *C. pneumoniae* and *B. pertussis* were also detected but their positive rates were much lower (Table 1). No statistically significant difference was found in the detection rates of overall URTI pathogens among the different age groups. Notably, FluA rate was significantly higher in the older adults of 45–65 years old (20.3%) but was significantly lower in the young children < 5 years old (6.8%) compared to other age groups (14.6–16.8%). Children < 5 years old also had much higher detection rates of hRV (11.4%), PIV (3.4%) and ADV (4.5%) (Table 1). In addition, we found RSV detection rate was significantly lower in the young adults of 18–45 years (2.1%) compared to other age groups (5.1–6.8%, excluding age group of 5–18 years due to only 1 positive case) (Table 1).

**Table 1:** Prevalence of URTI pathogens among different age groups

Age(years)	Case	Positive(%)	FluA(%)	FluB(%)	RSV(%)	hRV(%)	PIV(%)	ADV(%)	hMPV(%)	M.p(%)	C.P(%)	B.p(%)
0–5	88	31 (35.2)	6 (6.8)*	1 (1.1)	6 (6.8)	10 (11.4)***	3 (3.4)***	4 (4.5)***	1 (1.1)	3 (3.4)	0 (0.0)	0 (0.0)
5–18	95	30 (31.6)	16 (16.8)	3 (3.2)	1 (1.1)	2 (2.2)	2 (2.2)	1 (1.1)	1 (1.1)	7 (7.4)	1 (1.0)	0 (0.0)
18–45	563	162 (28.8)	82 (14.6)	25 (4.4)	12 (2.13)*	15 (2.7)	2 (0.4)	9 (1.6)	2 (0.4)	35 (6.2)	0 (0.0)	2 (0.4)
45–65	331	108 (32.6)	67 (20.3)***	7 (2.1)	17 (5.1)	11 (3.3)	0 (0.0)	1 (0.3)	3 (0.9)	14 (4.2)	0 (0.0)	0 (0.0)
> 65	144	41 (28.5)	21 (14.6)	3 (2.1)	8 (5.6)	3 (2.9)	3 (2.9)	0 (0.0)	4 (2.8)	6 (4.2)	0 (0.0)	0 (0.0)
Total	1221	372 (30.5)	192 (15.7)	39 (3.2)	44 (3.6)	41 (3.4)	10 (0.8)	15 (1.2)	11 (0.9)	65 (5.3)	1 (0.1)	2 (0.2)
p(Pearson’s _X_^2^		*p* = 0.599	*p* = 0.025*	*p* = 0.210	*p* = 0.02*	*p* = 0.001**	*p* = 0.003**	*p* = 0.012*	*p* = 0.106	*p* = 0.480		

Significant difference between a specific age group vs. all other age groups was identified using Pearson’s _X_^2^ TEST. *p < 0.05; **p < 0.01;***p < 0.001

Note: age groups with case # < 2 were excluded from the analysis

Abbreviations: *FluA* Influenza Virus A, *FluB* Influenza Virus B, *RSV* Respiratory Syncytial Virus, *hRV* Human Rhinovirus, *PIV* Parainfluenza Virus, *ADV* Adenovirus, *hMPV* Human Metapneumovirus, *M.p Mycoplasma pneumoniae*, *C.p Chlamydophila pneumoniae*, *Bp Bordetella pertussis*

Among all the cases positive for at least one URTI pathogens, the majority (81.7%) were of viral only infections. Only 11.6% of cases were of bacterial-only (*M. pneumoniae, C. pneumoniae*, *or B. pertussis*) infections and 6.7% of cases were of viral + bacterial co-infections (Table 2). Co-infections were quite common and found in 12.1% of the positive cases with the majority double co-infections and only 3 cases of triple-infections. No statistically significant difference was found among different age groups regarding the detection rates of different pathogen classification (viral vs bacterial, double vs triple infections) (Table 2).

**Table 2:** Classification of URTI pathogens among different age groups

Age(years)	Positive Case	Viral Only(%)	Viral + Bacterial(%)	Bacterial only(%)	Co-infection(%)	Double infection(%)	Triple infection(%)
0–5	31	28 (90.3)	1 (3.2)	2(6.5)	2(6.5)	1 (3.2)	1 (3.2)
5–18	30	22 (73.3)	3 (10.0)	5(16.7)	4(13.3)	4(13.3)	0 (0.0)
18–45	162	125 (77.2)	13 (8.0)	24(14.8)	21(13.0)	20(12.3)	1 (0.6)
45–65	108	94 (87.0)	5 (4.6)	9(8.3	12(11.1)	12(11.1)	0 (0.0)
> 65	41	35 (85.4)	3 (7.3)	3(7.3)	6(14.6)	5(12.2)	1 (2.4)
total (%)	372	304 (81.7)	25 (6.7)	25(6.7)	45(12.1)	42(11.3)	3 (0.8)
p (Pearson’s _X_^2^)		*p* = 0.107	*p* = 0.674	*p* = 0.281	*p* = 0.838	*p* = 0.671	*p* = 0.293

Significant difference between a specific age group vs. all other age groups was identified using Pearson’s _X_^2^

**Fig 1:**
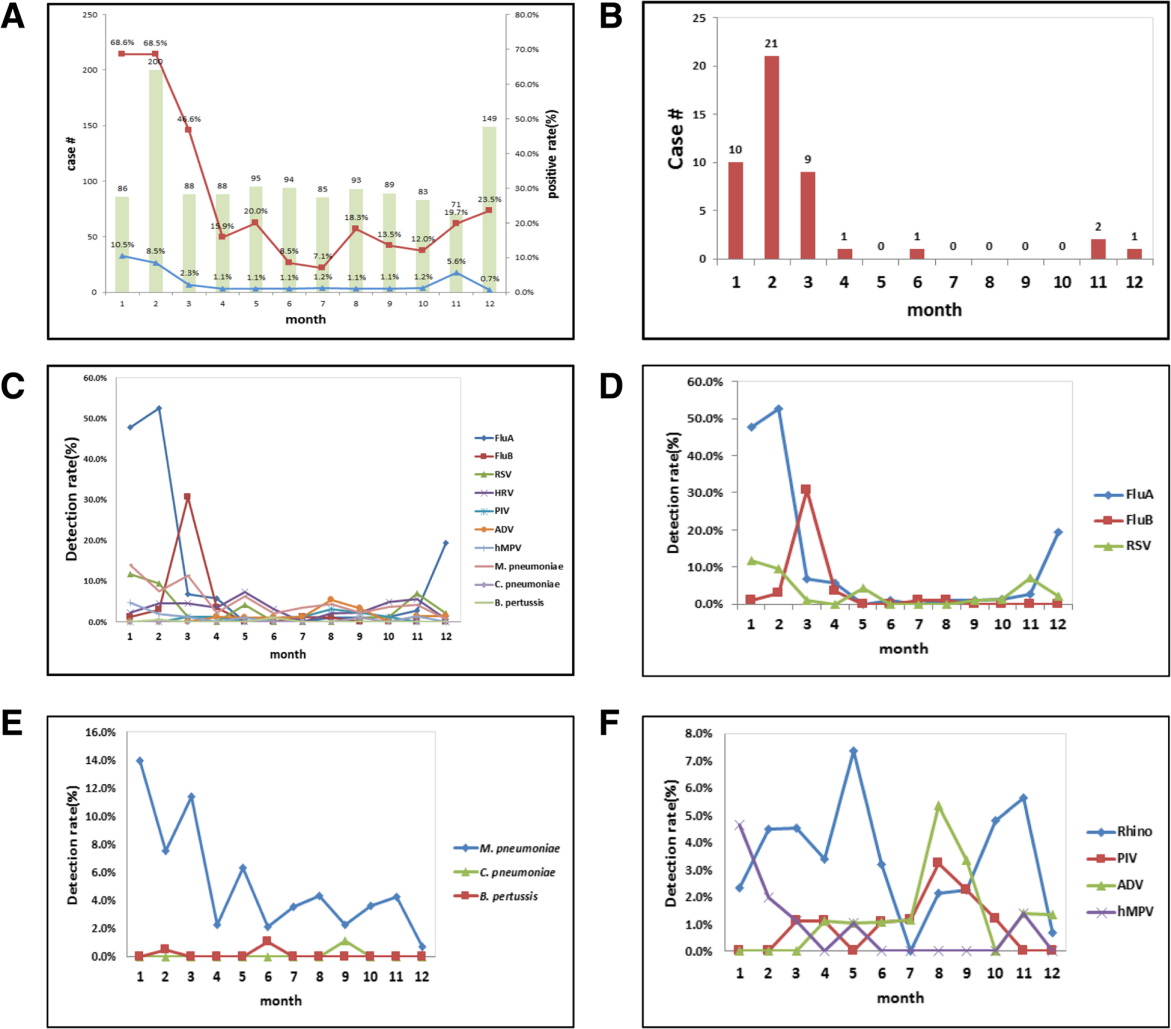
**a** Seasonal prevalence of combined URTI pathogens (red dot) and *S. pneumoniae* colonized in the upper respiratory tract (blue dot). **b** Seasonal prevalence of co-infections, defined by the detection of 2 or more than 2 URTI pathogens. **c** Seasonal prevalence of all URTI pathogens tested in this study. **d** Seasonal prevalence of FluA, FluB and RSV. **e** Seasonal prevalence of *M. pneumoniae, C. pneumoniae,* and *B. pertussis.***e** Seasonal prevalence of hRV, PIV, ADV and hMPV. Abbreviations: FluA Influenza Virus A, FluB Influenza Virus B, RSV Respiratory Syncytial Virus, hRV Human Rhinovirus, PIV Parainfluenza Virus, ADV Adenovirus, hMPV Human Metapneumovirus

During our 12-months study period in Shaoxing in 2016, an average of around 100 respiratory samples in each month were collected (Fig. 1 A). The prevalence of overall URTI pathogens was significantly higher in the months of January to March (46.6–68.5%) than the rest months of the year (7.1–23.5%) (Fig. 1A). Interestingly, the frequency of *S. pneumoniae* colonization also peaked in the months of January and February (8.5–10.5%) but stayed low in the rest of the months (0.7–2.3%) except November (5.6%). The overlapped peaks of the overall URTI pathogens and *S. pneumoniae* colonization frequency suggested there might be a positive correlation between the two. Co-infection rate also peak from January to March, overlapping with the peak of overall URTI pathogens (Fig. 1B). The seasonal pattern of the overall URTI pathogens was mainly attributed to the seasonal pattern of a few high prevalent URTI pathogens (Fig. 1C) including FluA, FluB, RSV (Fig. 1D) and *M. pneumoniae* (Fig. 1E), whose prevalence generally peaked from January to March. Other URTI pathogens seemed to have less obvious seasonal patterns, except for ADV and PIVs which appeared to both peak in the month of August (Fig. 1F).

**Fig. 2:**
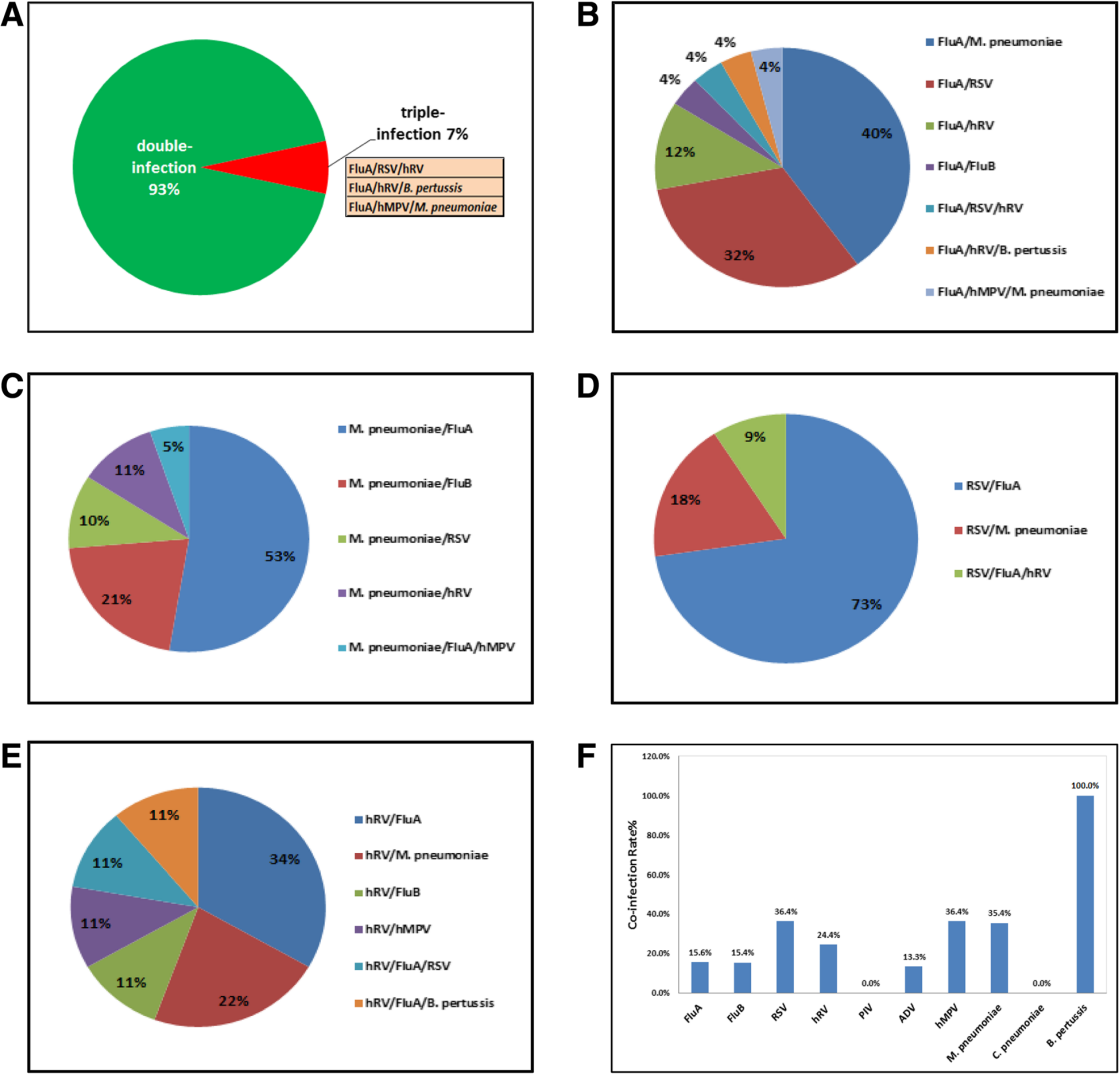
**a** Distribution of double-infection rate vs. triple infection rate. **b** Distribution of URTI pathogens co-infected with FluA. **c** Distribution of URTI pathogens co-infected with *M. pneumoniae*. **d** Distribution of URTI pathogens co-infected with RSV. **e** Distribution of URTI pathogens co-infected with hRV. **f** Co-infection rate in samples with each URTI pathogen detected. Abbreviations: FluA Influenza Virus A, FluB Influenza Virus B, RSV Respiratory Syncytial Virus, hRV Human Rhinovirus, PIV Parainfluenza Virus, ADV Adenovirus, hMPV Human Metapneumovirus

Respiratory infection is often characterized by mixed infection of various pathogens. In the 372 URTI pathogen positive samples, 45 (12.1%) were positive for two or more pathogens. The majority (93%) of the co-infections involved 2 pathogens. Only 3 triple-infection cases, all with FluA involved, were found (Fig. 2 A). Among the 45 co-infection cases, 66.7% (30/45) had FluA, 51.1% (23/45) had *M. pneumoniae*, 35.5% (16/45) had RSV, and 22.2% (10/45) had hRV. The most common co-infection was found to be FluA + *M. pneumoniae*, which accounted for 1.1% (13/1221) in all the samples and 3.5% (13/372) in the positive samples. The second most frequent co-infection was FluA + RSV, which accounted for 0.6% (7/1221) in all the samples and 1.9% (7/372) in the positive samples. All co-infection cases involved at least one of the top 5 high prevalent pathogens including FluA, FluB, RSV, hRV and *M. pneumoniae* (Fig. 2B-E), except for one case of ADV + *B. pertussis* co-infection. The co-infection rate broken down to each individual URTI pathogen was found to be very high in RSV (36.4%), hMPV(36.5%), and *M. pneumoniae* (35.4%) infections, and modestly high in hRV(24.4%), FluA (15.6%), FluB (15.4%) and ADV(13.3%) (Fig. 2F). The two cases of *B. pertussis* infections were both mixed infections with other viral pathogens.

**Fig. 3:**
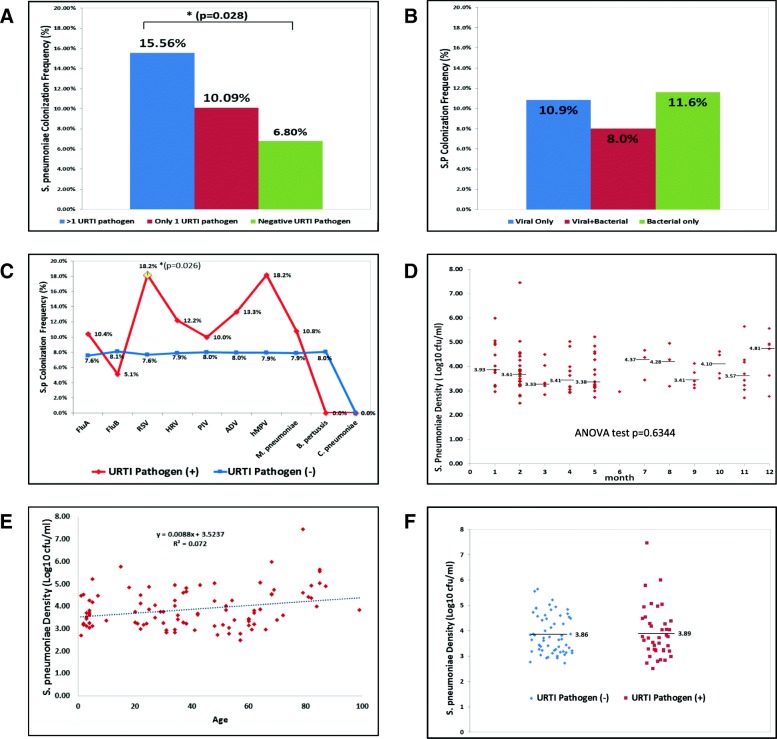
**a** Comparison of *S. pneumoniae* colonization frequency among 3 groups based on how many URTI pathogens were detected. **b** Comparison of *S. pneumoniae* colonization frequency among 3 groups based on the type of URTI pathogens. **c** Comparison of *S. pneumoniae* colonization frequency between positive samples and negative samples for each URTI pathogen. **d** Comparison of *S. pneumoniae* colonization density among samples collected in different month. **e** Correlation analysis of *S. pneumoniae* colonization density with age. **f** Comparison of *S. pneumoniae* colonization density between samples with and without URTI pathogen detected. Abbreviations: FluA – Influenza Virus A; FluB – Influenza Virus B; RSV: Respiratory Syncytial Virus; hRV: Human Rhinovirus; PIV: Parainfluenza Virus; ADV: Adenovirus; hMPV – Human Metapneumovirus

Among the patients with fever and flu-like symptoms, although the overall *S. pneumoniae* colonization frequency was 8.0% (98/1221) (Table 3), in most of the months except for January, February, March and November, the frequency was much lower (0.7–1.2%) (Fig. 1A). We found significantly increased *S. pneumoniae* colonization frequencies in both young children < 5 years old (21.6%) and the elderly > 65 years old (12.5%) compared to other age groups (4.2–7.3%) (Table 3). To investigate the relationship between *S. pneumoniae* colonization and URTI pathogens, we found there is a dose-dependent relationship between how many URTI pathogens were detected and the *S. pneumoniae* colonization frequency (Fig. 3 A). In cases without URTI pathogen detected, the *S. pneumoniae* colonization frequency was 6.8%, which increased to 10.1% when only one URTI pathogen was detected. This difference was with only borderline statistical significance (*p* = 0.061). However, in cases when more than one URTI pathogens were detected, the *S. pneumoniae* colonization frequency increased to 15.6% with a statistical significance (*p* = 0.028) (Fig. 3A). The increase of *S. pneumoniae* colonization frequency was found to be unrelated to the classification of the URTI pathogens since no statistically significant differences of *S. pneumoniae* colonization frequency were found among the case groups with viral-only, bacterial-only or bacterial + viral infections (Fig. 3B). To single out which URTI pathogen may contribute to this increase, we found although most URTI pathogens (except FluB, *B. pertussis* and *C. pneumoniae*) seem to be associated with higher S*. pneumoniae* colonization, the only statistically significant contributor was RSV infection (*p* = 0.026) (Fig. 3C). Without RSV infection, the *S. pneumoniae* colonization frequency was only 7.6%. With RSV infection, the frequency increased to 18.2%, more than 2 folds compared to the average *S. pneumoniae* colonization frequency (8.0%).

**Table 3:** Comparison of S. pneumoniae frequency and density among different age groups

Age(years)	*S. pneumoniae* Frequency(%)	*S. pneumoniae* Density (log10 cfu/ml)
0–5	19 (21.6)***	3.7 ± 0.6
5–18	4 (4.2)	4.6 ± 0.9
18–45	33 (5.9)	3.7 ± 0.7
45–65	24 (7.3)	3.5 ± 0.7
> 65	18 (12.5)	4.7 ± 1.0***
Total	98 (8.0)	3.9 ± 0.9

*Significant difference between a specefec age group vs. all other age groups was identified using Pearson’s _X_^2^ test for S. *pneumoniae* frequency density

*p < 0.05; **p < 0.01; ***p < 0.001

The age group of 5–18 is excluded for the analysis due to very low number of cases

The bacterial density of *S. pneumoniae* was found to be generally low (mean = 3.9 Log10 cfu/ml) in our patient population. The majority (90.8%, 89/98) had *S. pneumoniae* density lower than 5.0 Log10 cfu/ml (Fig. 3A). Only one sample had a very high *S. pneumoniae* density (7.5 Log10 cfu/ml), which is higher than 6.9 Log10 cfu/ml, a density cut-off shown to be able to predict streptococcal pneumonia in children aged < 5 years by a recent study [13]. The patient, however, was a 79-year-old elderly who was co-infected with RSV and hMPV. Due to the unavailability of clinical information, whether this patient had later developed streptococcal pneumonia was unknown. No significant difference of *S. pneumoniae* colonization density was identified among different months of the year (Fig. 3D). The correlation between *S. pneumoniae* colonization density and age as continuous intervals only showed a slight uptrend without statistical significance (Pearson correlation coefficient R^2^ = 0.072) (Fig. 3E). However, when the *S. pneumoniae* density was compared among different age groups, we found a statistically significant higher density (4.7 Log10 cfu/ml) in the elderly (> 65 years) compared to other age groups (3.5–3.7 Log10 cfu/ml, excluding age group of 5–18 due to only 4 positive cases), with more than 1-log difference (Table 3). The relationship between *S. pneumoniae* colonization density and URTI pathogens, however, was not observed since we did not find any difference in the *S. pneumoniae* density between the URTI pathogen positive cases vs. negative cases (Fig. 3F).

Only two cases (0.02%) of pertussis were found among the 1221 samples in 2016. Interestingly, both samples were also positive for other URTI pathogens. One sample was positive for *B. pertussis* and ADV, and the other sample was positive for *B. pertussis*, FluA and hRV. Both samples were from relatively young adults with age 20 and 30, who are in the child-bearing age and may transmit *B. pertussis* to their newborn babies. Since *B. pertussis* infection alone generally do not cause fever [14], our sample collection criteria might have resulted in a significant underestimation of the *B. pertussis* infection prevalence among the community in Shaoxing.

## Discussion

Several molecular epidemiological studies with the focus on hospitalized children have been reported in China, which demonstrated the complexity and diversity of pathogens involved in URTIs [15–18]. Although our overall findings were similar, our study was unique in a few aspects: 1) we did not set age limit, which provided a better representation of the whole community; 2) we only included outpatients with fever and flu-like symptoms which largely limited the cases to URTIs only; 3) we tested both viral pathogens and bacterial pathogens that cause URTIs; 4) we also investigated *S. pneumoniae* frequency and density in the upper respiratory tract in order to study the relationship between the URTI pathogens and *S. pneumoniae* colonization.

Consistent with other studies [15–18], we found FluA (15.7%), FluB (3.2%), RSV (3.6%), hRV (3.4%), and M. pneumoniae (5.3%) to be the most common URTI pathogens in patients with fever and flu-like symptoms. The majority (81.7%) of the URTIs were of exclusive viral etiology, and only 18.3% were of bacterial etiology (bacteria alone or bacteria + viral co-infections). These findings added to a large body of evidence supporting the guidelines that recommend against antibiotics prescription to treat URTIs without accurate diagnosis [3]. The seasonality pattern of most high-prevalence pathogens including FluA, FluB, RSV, and *M. pneumoniae* largely overlapped, with much higher prevalence in winter and spring (January – April). The mechanisms for the seasonality of the respiratory pathogens remain unclear but a few possible driven factors have been suspected such as enhanced wintertime survival of pathogens due to lower temperature and lower humidity, increased travel and social gathering due to more holidays in winter, as well as weakened immunity associated with a lack of vitamin D [19]. Interestingly, the prevalence of the overall URTI pathogens and the *S. pneumoniae* colonization frequency both peaked in the months of January and February, suggesting a possible positive correlation between URTI infections and *S. pneumoniae* colonization frequency.

The most significant finding in this study is that we discovered a pathogen variety-dependent positive correlation between how many URTI pathogens were detected and *S. pneumoniae* colonization frequency. Mixed infections with more than one URTI pathogens seemed to be associated with a significant increase of *S. pneumoniae* colonization frequency (from 6.8 to 15.6%). In addition, we identified RSV infection as the most significant contributor to the increased *S. pneumoniae* colonization frequency. The relationship between RSV infection and *S. pneumoniae* colonization have been recognized before. Studies have shown that RSV could enhance both the infectivity and virulence of *S. pneumoniae* [20, 21]. On the other hand, *S. pneumoniae* was found to enhance RSV infection both in vivo and in vitro [22]. *S. pneumoniae* colonization has also been linked to increased severity in children with RSV infections [23]. It should be noted that in our study, RSV is also associated with a higher percentage of co-infections (36.4%) compared to other URTI pathogens, which might be a confounding factor since we have demonstrated mixed infections itself could be associated with increased *S. pneumoniae* colonization frequency. Notably, our findings are novel as most other studies about the relationship between *S. pneumoniae* colonization and URTIs were limited to pediatric patients and viral infections, while our study included both viral and bacterial pathogens in patients of all ages. The biological mechanisms for the positive correlation between the *S. pneumoniae* colonization frequency and URTI pathogens require further investigation, which may help better understand the disease progression of URTIs and provide useful information for developing proactive monitoring protocols for high-risk patients.

The density (bacterial load) of the colonizing *S. pneumoniae*, however, was almost the same in our patients with or without detectable URTI pathogens, and there was no seasonal difference. These results indicate a lack of relationship between the *S. pneumoniae* colonization density and the URTI pathogens in patients only with URTIs. This is consistent with other studies showing that heavy *S. pneumoniae* colonization density is usually associated with more advanced lower respiratory streptococcal infections [10, 24]. However, we did find heavier density of *S. pneumoniae* colonization among elderly patients (> 65 years) regardless of detectable URTI pathogens, which combined with our finding that the *S. pneumoniae* frequency was also increased in this age group, provided consistent evidence to help explain why elderly patients are at higher risk of developing streptococcal pneumonia.

Importantly, we detected *B. pertussis* in 2 samples, both of which were also positive for other URTI viruses. This is particularly interesting because we only included patients with fever, which is not typically associated with *B. pertussis* infection [14]. Therefore, we might detect only a small fraction of *B. pertussis* cases with viral co-infections that presented with fever. The true prevalence of *B. pertussis* infection in this Chinese community is most likely underestimated. This is alarming since both *B. pertussis* positive cases were young adults in their childbearing age, who might transmit to their newborns. Although the vaccination coverage rate in China is estimated to be as high as 99%, surprisingly, 9% of the 24.1 million pertussis cases globally in 2014 were from China [25]. This is probably attributed to the shorter duration of protection by the acellular pertussis vaccines, which were introduced to China in 1995 [26] and had largely replaced the whole-cell pertussis vaccines since 2008 [27]. Recent studies have shown that the pertussis toxin IgG (a marker for the immunity) seropositive rate among the Chinese populations was as low as only 33% [28] and the misdiagnosis rate for pertussis in some areas was as high as 94.7% [29], highlighting the underrecognized severity of pertussis infections in China, and an urgency for improved diagnostic capacity, better surveillance system and vaccination strategies [30]. We plan to expand our molecular epidemiological study by using more appropriate criteria to include afebrile patients with prolonged non-productive cough [31, 32].

Our studies have several limitations. First, we did not detect coronaviruses, which are an important group of URTI pathogens and their prevalence can be as high as 5% in China [16]. However, this is not expected to change the conclusions that viral etiology dominated the URTIs, and *S. pneumoniae* colonization frequency increased in patients with detectable URTI pathogens. Second, we only included patients with fever, which may not be presented in many URTIs. The exclusion of afebrile patients could potentially lead to an underestimation of the true prevalence. Third, we did not test other pathogens including enterovirus, Epstein-Barr virus, cytomegalovirus and Group A *Streptococcus* that could also present with fever and flu-like symptoms [3, 33], which may partially explain the low positive rate during summer and fall. Fourth, we did not acquire the information about patient’s antibiotic usage, which may impact the prevalence of bacterial pathogens as well as the density of *S. pneumoniae* in the upper respiratory tract*.* Last, we did not include a control group of healthy people as a comparison. It has been shown that asymptomatic carriage of respiratory viruses and *M. pneumoniae* are not uncommon, especially in children [34–36]. We plan to expand our study to survey more people in the community including both symptomatic and asymptomatic individuals.

In summary, our molecular epidemiological study in a medium-sized city in Eastern China demonstrated that the pathogens causing the URTIs in the community were very diverse, complex, and dominated by viral infections. Co-infections were common and mainly involved the high frequency pathogens (FluA, RSV, hRV and *M. pneumoniae*). The seasonal patterns of the four most frequent pathogens (FluA, FluB, RSV and *M. pneumoniae*) overlapped and peaked during winter and spring (January to April), which also overlapped with the seasonal pattern of *S. pneumoniae* colonization in the upper respiratory tract. We also found a positive correlation between the *S. pneumoniae* colonization frequency (but not the density) and the number of URTI pathogens detected, in a pathogen variety-dependent manner. We observed higher *S. pneumoniae* colonization frequency in both the young children and the elderly, and higher *S. pneumoniae* colonization density in the elderly, regardless of whether URTI pathogens were detected or not. In addition, we found the majority of patients with URTIs had low *S. pneumoniae* colonization frequency and density, which is not indicative of Streptococcal lower respiratory infections.

## Conclusions

Our results strongly support the recommendation by the guidelines not to treat URTIs without accurate diagnosis, nor to use antibiotics for prophylaxis in patients with URTIs but without the signs and symptoms of pneumonia [3, 37]. Alarmingly we found 2 cases of pertussis in two young adults both of which had viral co-infections. Our fever criteria could potentially lead to a serious underestimation of the true pertussis prevalence in the studied community. A better pertussis epidemiological study aiming at afebrile patient with prolonged non-productive cough should be taken to assess the true prevalence of pertussis, which have been shown to be on the rise in China and requires immediately attention. The diversity and complexity of the URTI pathogens detected in this Chinese community also highlighted the need to improve the diagnostic capacity for URTIs, particularly by using more molecular testing, to encourage a more evidence-based antibiotics prescription practice and to alleviate the drug resistance burden caused by a massive scale of antibiotic abuse and misuse in China.

## Abbreviations

ADV: adenovirus
ANOVA: analysis of variance
*B. pertussis*: *Bordetella pertussis*
*C. pneumoniae*: *Chlamydophila pneumoniae*
Flu: influenza viruses
hMPV: human metapneumovirus
hRV: human rhinovirus
LOD: lower limit of detection
LOQ: lower limit of quantification
LRTIs: lower respiratory tract infections
*M. pneumoniae*: *Mycoplasma pneumoniae*
PIVs: parainfluenza viruses
RSV: respiratory syncytial virus
*S. pneumoniae*: *Streptococcus pneumoniae*
URTIs: upper respiratory tract infections
